# Hemodialysis-related changes in phenotypical features of monocytes

**DOI:** 10.1038/s41598-018-31889-2

**Published:** 2018-09-18

**Authors:** Vassilios Liakopoulos, Andreas Jeron, Aneri Shah, Dunja Bruder, Peter R. Mertens, Xenia Gorny

**Affiliations:** 1 Clinic of Nephrology and Hypertension, Diabetes and Endocrinology, Otto-von-Guericke-University Magdeburg, Leipziger Str. 44, 39120 Magdeburg, Germany; 2 Division of Nephrology and Hypertension, 1st Department of Internal Medicine, AHEPA Hospital, School of Medicine, Aristotle University of Thessaloniki, Thessaloniki, Greece; 3 Infection Immunology, Institute of Medical Microbiology, Infection Control and Prevention, Otto-von-Guericke University, Magdeburg, Germany; 4 Immune Regulation, Helmholtz Centre for Infection Research, Braunschweig, Germany

## Abstract

Hemodialysis (HD) patients exhibit chronic inflammation and leukocyte activation. We investigated the surface-marker profile of monocytes by flow cytometry to assess the chronic effect of uremia and the acute effect of dialysis on their phenotypical and functional features in 16 healthy controls (CON) and 15 HD patients before and after a polysulfone-based dialysis session. Median fluorescence intensities were analyzed indicating expression of CD14, CD16, integrins (CD11b, CD18), chemokine receptors (CCR2, CX3CR1), scavenger receptors (CD36, CD163) and Toll-like receptor-2 (TLR2). Before and after dialysis, HD patients harbour 0.9-fold less CD14^++^CD16^−^ (Mo1), 1.8-fold more CD14^++^CD16^+^ (Mo2) and CD14^+^CD16^++^ (Mo3) monocytes than CON. HD patients’ Mo1 showed elevated expression of CD11b (1.7-fold), CD18 (1.2-fold) and CD36 (2.1-fold), whereas CD163 expression was reduced in Mo1 and Mo2 (0.6-fold) compared to CON. These markers remained unaffected by dialysis. CX3CR1 expression on Mo2 and Mo3 was lower in HD patients before (0.8-fold) and further diminished after dialysis (0.6-fold). Stimulation of monocytes resulted in diminished responses in HD patients compared to CON. In conclusion, a systematic analysis of the expression of particular surface markers on distinct monocyte subsets may help to distinguish between uremia and/or dialysis induced effects and to evaluate the functionality of monocytes and biocompatibility of HD.

## Introduction

Patients with End Stage Renal Disease (ESRD) on hemodialysis (HD) exhibit a chronic inflammatory state with increased oxidative stress, endothelial dysfunction and impaired immune response with activation and/or senescence of peripheral blood mononuclear cells (PBMCs)^1–3^. The dialysis procedure *per se* adds acutely to these adverse conditions due to the exposure to synthetic materials of dialysis filters and tubing. In former times the use of “bio-incompatible”, complement-activating cellulose membranes reinforced PBMC and platelet activation^4^. Nowadays, efforts have been made to improve biocompatibility and solute removal capability of membranes made of synthetic polymers that reduce immunologic responses caused by the repetitive immediate contact between circulating leukocytes and dialysis membranes^5^. However, an HD session remains a highly proinflammatory and prooxidant procedure.

Monocytes are key players in systemic inflammation and may change their activation status in response to toxins, invading pathogens and contact to HD-related materials^6^. Based on the surface expression of the LPS co-receptor CD14 and the immunoglobulin Fcγ receptor type III CD16 monocytes may be classified into three subpopulations with phenotypical and functional differences^7,8^. Classical CD14^++^CD16^−^ Mo1 monocytes show highest expression of scavenger receptors CD36 and CD163, suggesting a function in clearance of apoptotic cells, microparticles, modified proteins, lipids, lipoproteins and haptoglobins derived from lysed erythrocytes^8^. Intermediate CD14^++^CD16^+^ Mo2 monocytes express high levels of Toll-like receptors (TLRs) and molecules essential for antigen presentation^8^. By expressing high levels of the chemokine receptor CX3CR1, non-classical CD14^+^CD16^++^ Mo3 monocytes patrol the vessel walls and may firmly adhere to the endothelium^8^. Especially Mo2 and Mo3 monocytes release proinflammatory cytokines like interleukin (IL)-1, IL-6 and TNFα in response to pathogenic stimuli^8,9^. It has been described that HD patients contain more CD16^+^ monocytes compared to healthy controls, suggesting a prevailing proinflammatory profile^10,11^.

Mo1 monocytes are believed to mature into non-classical Mo3 monocytes *via* the intermediate Mo2 state^7,12^. In response to diverse pathogenic stimuli they may undergo phenotypic changes in form of an altered expression of surface receptors^7,8,12^. Therefore, the assessment of surface marker expression might serve as an estimate of the inflammatory state of an individual. Besides CD14 and CD16 we have chosen a combination of seven surface markers, which were measured simultaneously. These include CD11b, CD18, CD36, CD163, CCR2, CX3CR1, and Toll-like receptor TLR2. CD18/CD11b integrins are exclusively expressed on leukocytes where they mediate cell-cell interactions and enable leukocytes *inter alia* to attach to the endothelium and migrate out of the bloodstream to an inflamed area^13,14^. The scavenger receptors CD36 and CD163 are involved in the clearance of cellular debris as well as released and modified cellular components such as oxidized lipoproteins^15^. The chemokine receptors CCR2 and CX3CR1 are particularly relevant for monocyte infiltration in the course of inflammation, oxidative stress and atherosclerosis^16–20^. Toll-like receptors (TLRs) recognize a divergent collection of pathogen-derived ligands, thereby activating their host cell, *inter alia* monocytes, which in turn respond with the production of proinflammatory cytokines^21^. For hemodialysis patients, available data on monocytic receptor expression are scarce and controversial and none of the precedent studies took a closer look at the distinct monocyte subpopulations in combination with assessing diverse surface markers^14,22–25^. Therefore, our aim is to provide an overall picture on monocytic phenotypes and changes related to uremia and the dialysis procedure.

## Results

### Study cohorts and methodology

To evaluate alterations in the steady-state and the influence of the dialysis procedure on phenotypic characteristics of monocytes, 15 patients on hemodialysis (HD) with a mean age of 64.1 ± 17.4 years (5 female, 10 male) and 16 healthy age and gender matched controls (CON, mean age of 55.8 ± 6.3 years, 5 female, 11 male) were enrolled in the study. A detailed description of both cohorts including absolute leukocyte and monocyte numbers and comorbidities is provided in Supplementary Table 1. 8-color flow cytometry and three different antibody panels were used to estimate the expression of surface markers before and after a dialysis session with a gating strategy for each panel outlined in Supplementary Fig. 1.

### Alterations of monocyte subpopulations in hemodialysis patients

**Figure 1:**
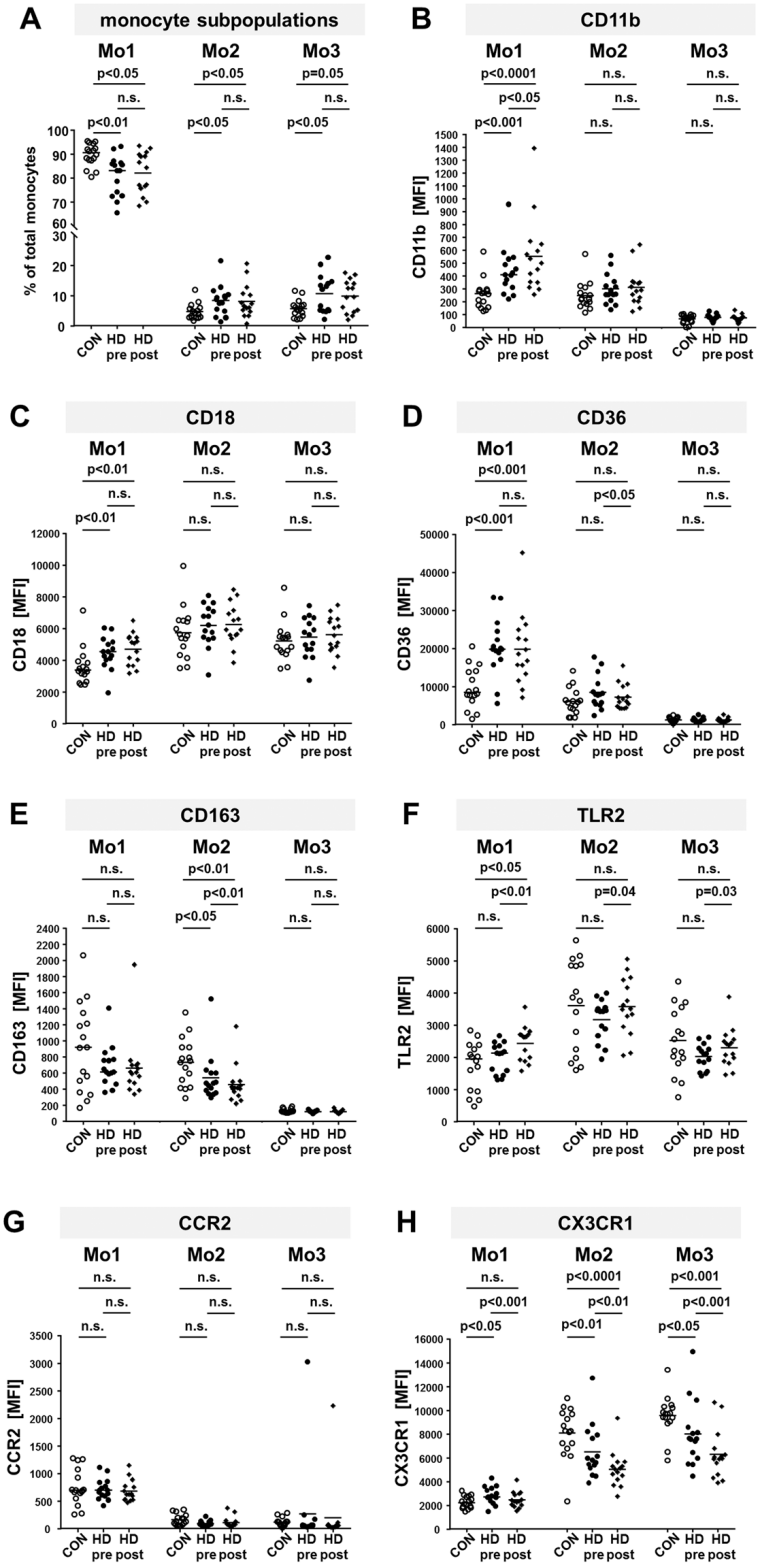
Comparison of percentages of monocyte subpopulations and expression of surface markers on monocytes from healthy donors and dialysis patients. Blood from healthy donors (CON) and hemodialysis patients (HD) before (pre) and after (post) a dialysis session was stained with fluorochrome labeled antibodies to gate for monocyte subsets as outlined in supplementary Fig. 1. The scatter plots show the percentages of Mo1, Mo2 and Mo3 of total monocytes (**A**), the expression of CD11b (**B**), CD18 (**C**), CD36 (**D**), CD163 (**E**), TLR2 (**F**), CCR2 (**G**) and CX3CR1 (**H**) from 16 healthy controls (blank circles) and 15 HD patients before (HD pre, black circles) and after (HD post, black diamonds) a dialysis session. P-values comparing CON with HD pre or HD post were calculated with Mann-Whitney U-test, p-values comparing HD pre and HD post with Wilcoxon matched-pairs signed rank test. MFI: median fluorescence intensity. n.s.: not significant.

HD patients exhibited an increased absolute number of monocytes per µl (HD: 661 ± 240, CON: 528 ± 179; p < 0.05) and an altered distribution of monocyte subsets due to a prevailing inflammatory state. They showed significantly less classical Mo1 monocytes (CON: 89.6 ± 4.5%, HD pre: 80.1 ± 8%, HD post: 82.1 ± 8.4%, CON/HD pre: p = 0.002, CON/HD post: p = 0.01) and more proinflammatory intermediate Mo2 (CON: 4.7 ± 2.5%, HD pre: 8.4 ± 4.8%, HD post: 8.1 ± 5.3%, CON/HD pre: p = 0.02, CON/HD post: p = 0.02) and non-classical Mo3 monocytes (CON: 5.8 ± 2.7%, HD pre: 10.7 ± 6.1%, HD post: 9.8 ± 5.2%, CON/HD pre: p = 0.05, CON/HD post: p = 0.04) compared to healthy volunteers both before and after a HD session (Table 1, Fig. 1A).

**Table 1:** Values of distribution of monocyte subpopulations and surface marker expression.

group	CON	HD pre	HD post	CON	HD pre	HD post	CON	HD pre	HD post
subpopulation	Mo1			Mo2			Mo3		
proportion [%]	89.6 ± 4.5	80.9 ± 8.1*	82.1 ± 8.4*	4.7 ± 2.5	8.4 ± 4.8*	8.1 ± 5.3*	5.8 ± 2.7	10.6 ± 6.1*	9.8 ± 5.2
CD11b [MFI]	257 ± 113	438 ± 175****	553 ± 283***^,#^	250 ± 104	299 ± 114	314 ± 139	67 ± 31	79 ± 22	74 ± 25
CD18 [MFI]	3598 ± 1122	4474 ± 994**	4693 ± 962**	5749 ± 1557	6209 ± 1275	6266 ± 1249	5226 ± 1191	5456 ± 1215	5606 ± 1091
CCR2 [MFI]	755 ± 315	715 ± 183	682 ± 188	161 ± 97	93 ± 48	110 ± 96	117 ± 77	270 ± 738	195 ± 544
CX3CR1 [MFI]	2289 ± 502	2858 ± 705*	2483 ± 639^###^	8113 ± 2063	6524 ± 2173**	5048 ± 1444****^,##^	9563 ± 1642	8010 ± 2599*	6289 ± 1952***^,###^
CD36 [MFI]	9725 ± 5205	20359 ± 7357***	19782 ± 9042***	6049 ± 3369	8490 ± 4254	7190 ± 3189^#^	1258 ± 577	1237 ± 548	1142 ± 558
CD163 [MFI]	910 ± 528	685 ± 249	663 ± 366	736 ± 286	542 ± 290*	457 ± 226**^,##^	131 ± 25	123 ± 14	122 ± 18
TLR2 [MFI]	1725 ± 732	1969 ± 457	2440 ± 514*^,##^	3604 ± 1365	3174 ± 612	3577 ± 858^#^	2521 ± 998	2016 ± 379	2299 ± 583^#^

The table displays monocyte proportions and median fluorescence intensities (MFI) of surface markers in monocyte subsets Mo1, 2 and 3 in healthy volunteers (CON) and hemodialysis patients (HD) before (pre) or after (post) a dialysis session. Asterisks and hashes indicate the power of significance between CON and HD or HD pre and post (*CON/HD p < 0.05, **CON/HD p < 0.01, ***CON/HD p < 0.001, ****CON/HD p < 0.0001, ^#^HD pre/post p < 0.05, ^##^HD pre/post p < 0.01^, ###^HD pre/post p < 0.001).

*CON/HD p < 0.05, **CON/HD p < 0.01, ***CON/HD p < 0.001, ****CON/HD p < 0.0001, ^#^HD pre/post p < 0.05, ^##^HD pre/post p < 0.01, ^###^HD pre/post p < 0.001.

### Integrin expression is upregulated in Mo1 monocytes of hemodialysis patients

Both CD11b and CD18 integrin subunits were significantly upregulated on Mo1 monocytes from HD patients compared to healthy controls (Table 1, Fig. 1B,C; CD11b CON/HD pre: p = 0.001, CON/HD post: p < 0.0001, HD pre/post: p = 0.02; CD18 CON/HD pre: p = 0.005, CON/HD post: p = 0.003, HD pre/post: p = 0.6). In contrast, no significant difference in CD11b or CD18 expression on Mo2 and Mo3 monocytes from dialysis patients compared to healthy controls was observed. After the dialysis procedure Mo1 CD11b expression was mildly enhanced, whereas there was no immediate effect on CD18 expression.

### Scavenger receptors on monocytes from HD patients

Our results show that CD36 was ~2-fold upregulated on Mo1 monocytes from HD patients compared to healthy controls (Table 1, Fig. 1D; CON/HD pre: p = 0.0002, CON/HD post: p = 0.0007). Due to maturation, Mo2 and Mo3 monocytes exhibit lower expression of CD36 and no difference between healthy volunteers and HD patients could be detected (Fig. 1D). CD163 may be shed from the surface upon activation of the cell. Meeting this expectation, CD163 surface expression was lower on Mo1 and Mo2 monocytes from HD patients compared to healthy subjects, although there is a large variability within both cohorts (Table 1, Fig. 1E; Mo1: CON/HD pre: p = 0.3, CON/HD post: p = 0.3; Mo2: CON/HD pre: p = 0.03, CON/HD post: p = 0.004, HD pre/post: p = 0.002). Mo3 monocytes do not express CD163 (Fig. 1E). Furthermore, the expression of both scavenger receptors is not influenced by the dialysis procedure.

### The dialysis procedure results in up-regulation of Toll-like receptor-2

**Figure 2:**
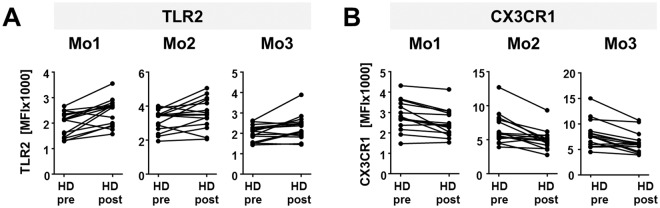
Personalized expression of CX3CR1 and TLR2 before and after dialysis. The graphs show the median fluorescence intensity (MFI) of CX3CR1 (**A**) or TLR2 (**B**) on Mo1, Mo2 and Mo3 monocytes from 15 individual patients before (pre) and after (post) hemodialysis (HD).

In this study, we exemplarily considered expression of TLR2 as a member of the TLR protein family. Before the dialysis session, only Mo2 and Mo3 monocytes from HD patients showed slightly decreased TLR2 levels compared to healthy volunteers. After dialysis this disparity was normalized compared to the healthy cohort, leading to a significant upregulation when comparing pre and post dialytic TLR2 expression levels (Table 1, Fig. 1F; Mo1: CON/HD pre: p = 0.4, CON/HD post: p = 0.02, HD pre/post p = 0.002; Mo2: CON/HD pre: p = 0.4, CON/HD post: p = 0.8, HD pre/post p = 0.04, Mo3: CON/HD pre: p = 0.2, CON/HD post: p = 0.6, HD pre/post p = 0.03). This is also reflected by individual patient data (Fig. 2A).

### Chemokine receptor CX3XR1 expression is decreased on Mo2 and Mo3 monocytes from HD patients

Only Mo1 monocytes exhibit a considerable level of CCR2^18^. Here, no difference of CCR2 expression between healthy donors and HD patients, neither before nor after a dialysis session, could be observed (Table 1, Fig. 1G). In contrast, significant differences of CX3CR1 expression could be found in all monocyte subpopulations (Table 1, Fig. 1H). Before dialysis its expression was increased on Mo1 monocytes from HD patients compared to healthy controls (CON/HD pre: p = 0.02, CON/HD post: p = 0.5, HD pre/post: p = 0.0003). This increase was ablated after dialysis. In Mo2 as well as Mo3 monocytes, CX3CR1 expression was strongly decreased in HD patients compared to the healthy cohort and the dialysis procedure even further reduced CX3CR1 expression (Mo2: CON/HD pre: p = 0.009, CON/HD post: p < 0.0001, HD pre/post: p = 0.003; Mo3: CON/HD pre: p = 0.02, CON/HD post: p = 0.0006, HD pre/post: p = 0.0009). Taking a closer look at the data of each individual patient reveals that CX3CR1 expression is uniformly downregulated after the dialysis procedure (Fig. 2B).

**Figure 3:**
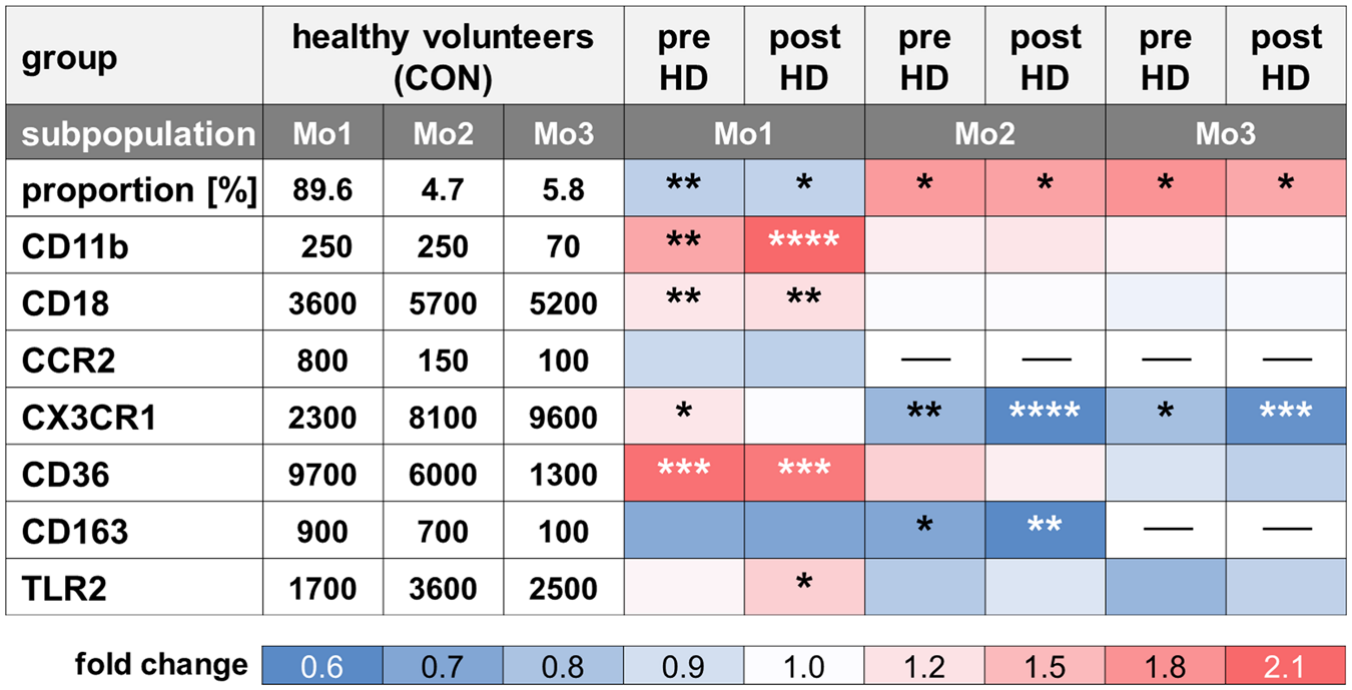
Summary of the findings. The table in style of a heatmap summarizes the changes of percentage or marker expression in the indicated monocyte subpopulation, whereby values obtained from hemodialysis patients (HD) before (pre) and after (post) dialysis were related to healthy volunteers (CON). Asterisks indicate the power of significance (*p < 0.05, **p < 0.01, ***p < 0.001, ****p < 0.0001); MFI: median fluorescence intensity.

Our findings for all investigated markers are summarized in a heatmap (Fig. 3).

### Uremic conditions strongly influence CX3CR1 expression on monocytes

**Figure 4:**
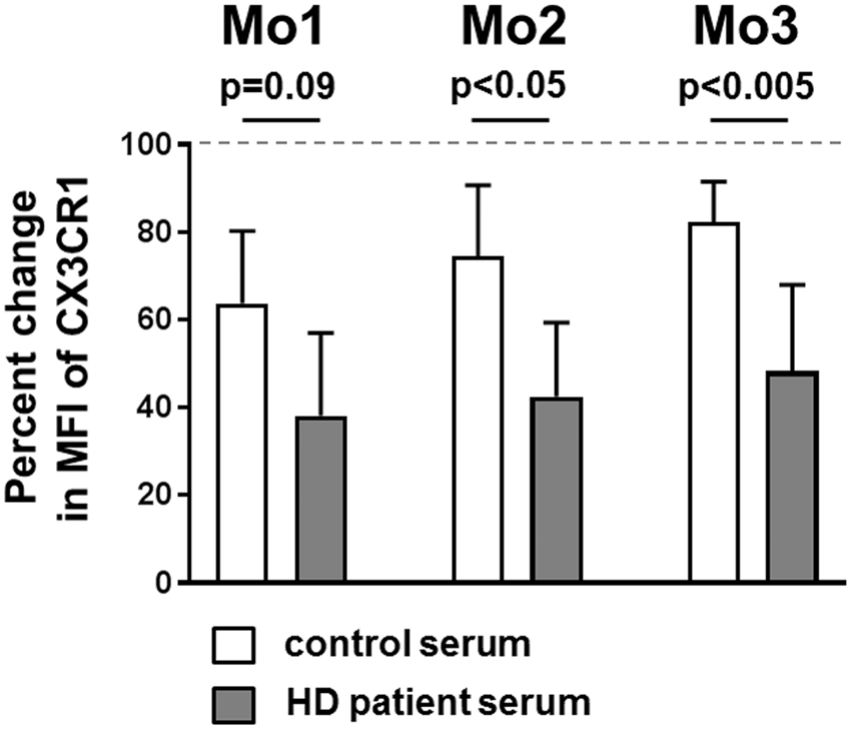
Uremic conditions strongly influence CX3CR1 expression on monocytes. Whole blood from healthy donors (blood group 0) was diluted 1:1 with self-serum (reference), non-uremic control or HD patient serum. (**A**) The diagram shows the percentual change of CX3CR1 surface expression on Mo1, Mo2 and Mo3 monocytes after 4 h incubation at 37 °C, 5% CO_2_ with the indicated foreign serum compared to incubation with self-serum (100%, indicated by a dotted horizontal line). P-values were calculated using the Mann-Whitney U-test.

To further assess the influence of uremic conditions on monocytes, whole blood from healthy volunteers was diluted 1:1 with non-uremic control or HD patient serum and incubated for 4 h. To account for the effect that cell culture materials might have on monocyte phenotypes, stimulation with self-serum in the same culture plate served as negative control. The distribution of monocyte subsets was not altered after stimulation with foreign control or HD patient serum (Supplementary Fig. 2). CD18 expression was enhanced in all monocyte subsets, CD36 was only enhanced on Mo1 monocytes and CD163 expression was attenuated on Mo1 and Mo2 monocytes exposed to foreign serum regardless of the presence of uremic substances, indicating a general low-grade activation of monocytes due to foreign components in their immediate vicinity (Supplementary Fig. 3). In contrast, after incubation with uremic serum CX3CR1 expression was more diminished on all monocyte subpopulations compared to incubation with non-uremic serum (Fig. 4; control/HD patient serum Mo1: p = 0.09, Mo2: p < 0.05, Mo3: p < 0.005). In conclusion, the expression of the chemokine receptor CX3XR1 is strongly influenced by the toxins present in uremic serum.

### After dialysis procedure leukocytes show decreased adhesion capacity

**Figure 5:**
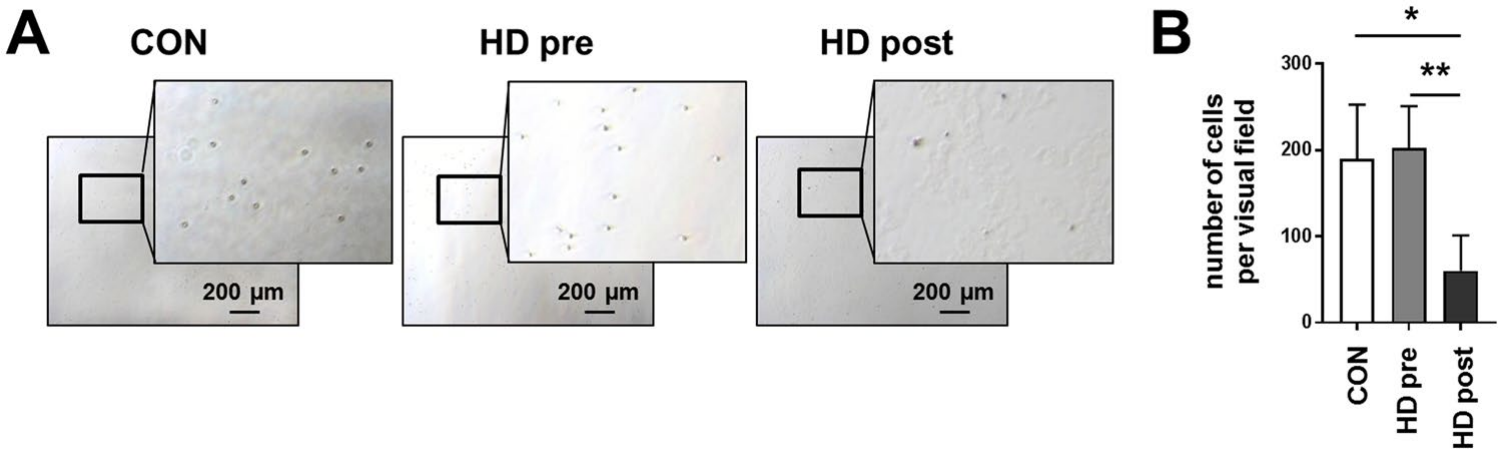
Adhesion capacity of leukocytes before and after dialysis Whole blood from healthy donors (n = 3) and HD patients (n = 5, collected either before or after a single dialysis session) was incubated for 30 min in cell culture plates. (**A**) Characteristic microscopic pictures taken with 5x magnification and enlargements of the framed region in each picture show adherent leukocytes of healthy volunteers (left), HD patients before (center) and after (right) a single dialysis session. The scale bar shows 100 µm. (**B**) The graph shows the number of adherent leukocytes from healthy volunteers (white bar), or HD patients before (grey bar) and after (black bar) a single dialysis session. P-values were calculated using the unpaired or paired t test (for CON/HD post or HD pre/HD post, respectively).

Because CX3CR1 mediates arrest and adhesion of CD16^+^ monocytes^26^, we wanted to test the effect of uremia and dialysis on leukocyte adhesion capacity. Whole blood was incubated in cell culture plates and cells that remained adherent to the plastic after thorough washing were counted. Leukocyte numbers were comparable among all three groups. Representative microscopic pictures are shown in Fig. 5A. Before dialysis, leukocyte adhesion was similar between healthy controls and dialysis patients (Fig. 5B). After the dialysis procedure there were significantly less adherent leukocytes per visual field in HD patients’ blood compared to controls and the same patients’ blood before the dialysis session (Fig. 5B; CON 190 ± 63, HD pre 203 ± 48, HD post 60 ± 41; CON/HD post p = 0.01, HD pre/HD post p = 0.003).

### LPS challenge leads to a differential response of HD patients’ monocytes

**Figure 6:**
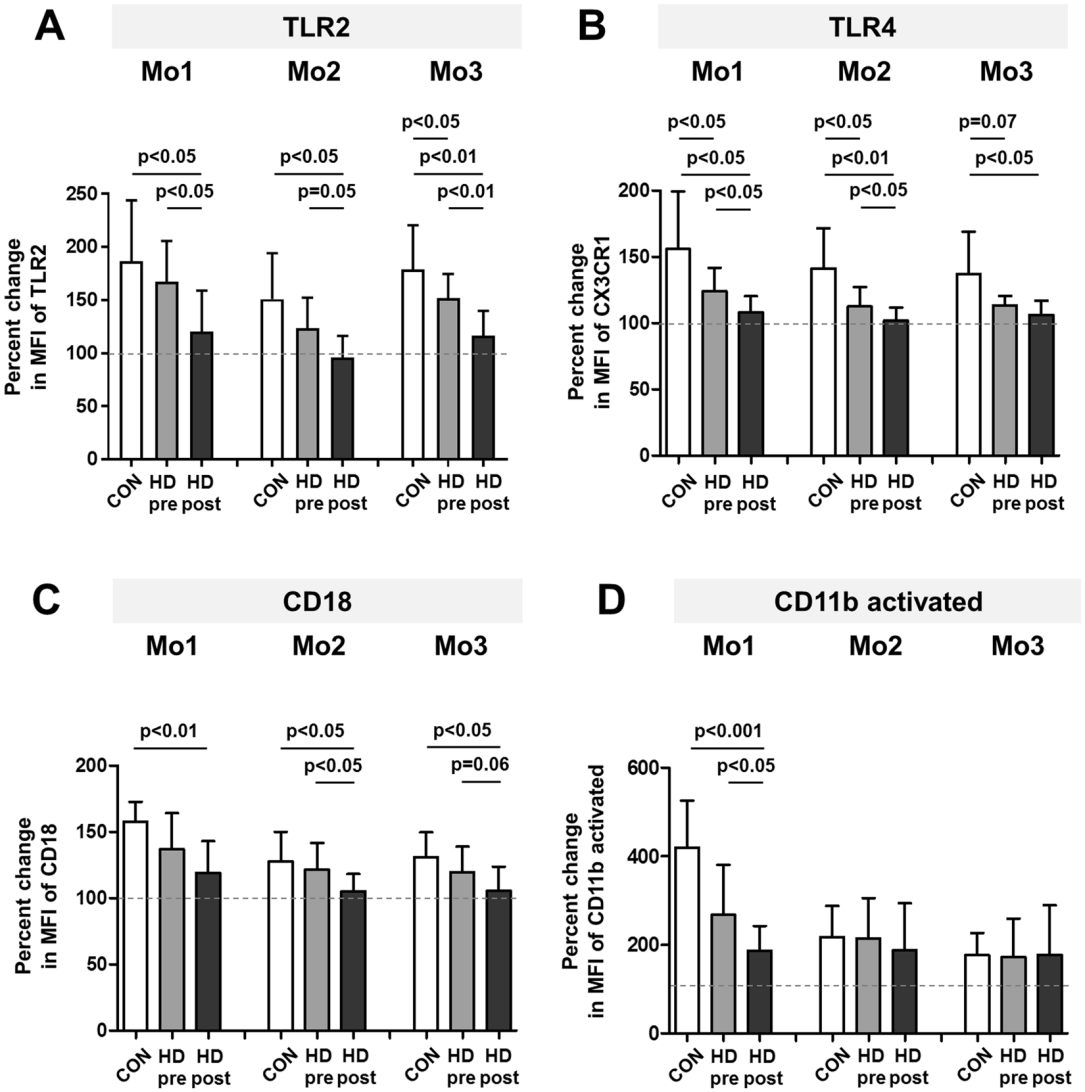
Response of healthy controls’ or HD patients’ monocytes upon LPS stimulation Whole blood from healthy donors (CON, n = 7) and HD patients (HD, n = 9, collected both before, pre, and after, post, a single dialysis session) was incubated for 30 min with 10 ng/ml LPS or left untreated. The graphs show the percentual change of TLR2 (**A**), TLR4 (**B**), CD18 (**C**) or CD11b activated (**D**) expression on all three monocyte subpopulations in healthy volunteers (white bars), or HD patients before (grey bars) and after (black bars) a single dialysis session. P-values comparing CON with HD pre or HD post were calculated with two-tailed Mann-Whitney U-test, p-values comparing HD pre and HD post with Wilcoxon matched-pairs signed rank test. MFI: median fluorescence intensity.

To assess whether altered expression of monocyte surface markers might impact or hint at altered monocyte functionality, we investigated the responsiveness of monocytes upon LPS challenge. The incubation of whole blood with 10 ng/ml LPS clearly increased the expression of TLR2, TLR4, CD18 and activated CD11b on all monocyte subpopulations (Fig. 6A–D). Notably, this increase was significantly stronger on monocytes from healthy controls compared to HD patients before dialysis. Moreover, for Mo2 and Mo3 subpopulations significant LPS-induced elevation of marker expression was absent after the dialysis procedure (Supplementary Fig. 4A–D). This indicates a reduced responsiveness of monocytes from HD patients, especially after the dialysis procedure, towards stimuli mimicking bacterial infection.

## Discussion

This study aims to provide a concise overview of the phenotypic features of monocytes in hemodialysis patients affected either by a chronic inflammatory state or acutely by the hemodialysis procedure.

One important finding of our study, which has been noted before, is a shift towards more proinflammatory CD16^+^ Mo2 and Mo3 monocytes in HD patients^10,27,28^. The increase in the frequency of CD16^+^ monocytes has been attributed to chronic inflammation accompanying ESRD and has been associated with increased mortality in HD patients^28,29^. Due to the accepted view of monocyte maturation, the presence of CD16 on the surface of monocytes can be considered a maturation marker^7^. There is a bulk of evidence linking CD16^+^ monocytes with infections, acute decompensated heart failure and (low grade) inflammation associated with metabolic disorders like insulin resistance, diabetes mellitus and hypercholesterolemia^30^. Furthermore, the role of different monocyte subsets in atherosclerotic lesions, coronary artery disease or cardiac remodeling after an acute coronary syndrome is under debate^31^.

A transient decrease in the number of total monocytes during a HD session has been well documented, but most studies agree that they return back to baseline levels at the end of a dialysis session^32,33^. A slower kinetic pattern of reappearance has been noted for CD16^+^ monocytes^34^. In our study, the HD procedure did not attenuate the skewed distribution observed in monocytic subpopulations, suggesting it to be a ‘regular’ characteristic of HD patients. This observation is further supported by previous studies that failed to show an acute effect of dialyzers with different biocompatibility profiles on the total number of CD16^+^ monocytes^32,35^.

We found increased expression of the integrin subunits CD18 and CD11b before and after hemodialysis treatment on classical Mo1 monocytes from HD patients. β2 integrins are leukocytic adhesion molecules that play an important role in the immune-inflammatory response^13^. CD18 represents the common β2 subunit in the integrin heterodimers, whereas the α-subunit is interchangeable and specific for a distinct leukocyte population. Monocytes mainly express the combination of CD18 and CD11b, also referred to as Mac-1. The latter confers binding to intercellular adhesion molecules ICAM-1 to -3 which are upregulated on activated endothelial cells and may be found at sites of increased oxidative stress and inflammation. Mac-1 may be stored in intracellular vesicles and emerge at the surface upon cell activation promoting enhanced adhesion to the endothelium and transmigration towards inflammatory sites^14^. A study of patients on cellulose-based hemodialysis found that CD11b expression on total leukocytes was only temporarily elevated after 15 min, returning to baseline after 2 h of dialysis^36^. Notably, this study did not distinguish between leukocyte populations. A later study, again with cellulose membrane dialyzers, reported on a significant increase in surface expression of CD11b and CD18 on granulocytes and monocytes of HD patients before dialysis, followed by a reduction right after a hemodialysis session^14^. In another study, in which low and high flux polysulfone dialyzers were employed, decreased monocytic levels of CD11b were found before the dialysis treatment. After dialysis, they were significantly increased compared to healthy controls^23^. In our study, monocytic expression of CD18 before dialysis was similar to the control group and only mildly enhanced after the dialysis procedure. Our patients were dialyzed with the newer polysulfone-based high-flux membrane Helixone (FX series, Fresenius Medical Care, Germany), which might explain the divergent results compared to the literature. Summarizing, our results point to a chronic inflammation-based phenotypic alteration of Mo1 monocytes, neither acutely enhanced nor abrogated by dialysis.

Monocytes are able to clear cell debris as well as extraneous or pathologically modified proteins, lipids or lipoproteins from the circulation *via* scavenger receptors^15^. Consequently, these receptors might be upregulated upon monocyte stimulation. We were able to show an overall two-fold increase in CD36 expression on Mo1 monocytes but not on Mo2 and Mo3 monocytes. An upregulation of CD36 on CD14^+^ monocytes was shown previously for patients with type II diabetes, attributable to hyperglycemia^37^. Later, CD36 mRNA and protein levels of PBMCs have been shown to be upregulated in type II diabetes patients, an indication of monocyte activation and a biomarker for a proinflammatory state^38^. In concordance with our results, Hernandez and coworkers found a significant upregulation of CD36 on total leukocytes in uremic patients before dialysis, which was not influenced during the dialysis procedure^36^. An upregulation of CD36 on CD14^+^ monocytes from HD patients has also been shown, although a distinction of monocyte subsets was lacking^24,25^. In contrast to CD36, CD163 expression is highly sensitive towards different chemicals used for anti-coagulation and the PBMC isolation procedure^22^. Highest CD163 levels could be obtained by staining whole blood anti-coagulated with EDTA, as applied in our study. By distinguishing Mo1, Mo2 and Mo3 monocytes, we could show a decrease in the surface expression of CD163 in Mo2 monocytes from HD patients compared to healthy controls. A similar trend, not reaching statistical significance, could be observed on Mo1 monocytes. The extracellular domain of CD163 can be shed from the surface of activated monocytes upon exposure to proinflammatory stimuli^39^. Therefore, decreased surface levels of CD163 are indicative of inflammatory conditions present in HD patients. Recently, Min *et al*. reported on a decrease in the percentage of CD163 positive monocytes in patients with diabetes suffering from complications (including renal disease) as compared to diabetics without complications^40^. Therefore, we compared diabetics and non-diabetics from our patient cohort without detecting significant differences between both subgroups (data not shown).

TLRs are expressed in a variety of immune and non-immune cells recognizing products of various pathogen-derived ligands. Contact between receptor and ligand activates monocytes leading to the release of proinflammatory cytokines^21^. Thus, potential alterations in TLR expression might substantially contribute to the increased systemic inflammation and susceptibility to infections observed in ESRD^41^. No statistical significance in the difference of monocytic TLR2 levels between healthy controls and dialysis patients before dialysis was reached due to the high degree of variation. However, after dialysis we observed a clear increase in monocytic TLR2 expression especially on Mo1 monocytes. Available data from other groups regarding the expression of TLRs on monocytes of ESRD patients are few and controversial. In stage 5 predialysis chronic kidney disease (CKD) patients a reduced expression of TLR4 has been observed. This positively correlated with cytokine synthesis and was more pronounced in patients with a history of repeated infections^42^. A decreased expression of TLR4 has also been found in hemodialysis patients, correlating with hemodialysis vintage, irrespective of different types of dialyzer membranes. The same study showed a decreased expression of TLR2 in patients on peritoneal dialysis^43^. On the contrary, another study reported that TLR2 and TLR4 were upregulated on monocytes and polymorphonuclear leukocytes in patients undergoing hemodialysis with cellulose triacetate dialyzers compared to healthy controls^41^. TLR4 was also found to be upregulated on CD14^dim^ monocytes in ESRD patients undergoing hemodialysis with a low flux dialyzer^44^. In another study, TLR2 expression on CD14^+^ monocytes of HD patients exposed to low flux polysulfone dialyzers was increased compared to matched healthy controls^45^. A transient decrease occurred after 120 min of dialysis treatment but post-dialytic levels returned to baseline. In contrast to TLR2, the MFI of TLR4 was initially not altered but increased 24 h after the dialysis session. The main difference between other studies and ours is the specific characteristics and type of the dialyzer membrane, which might explain the disparity between the results, which in our case show that monocytes are acutely affected by the dialysis procedure.

The interaction between the chemokines fractalkine/CX3CL1 and monocyte chemoattractant protein-1 (MCP-1)/CCL2 with their respective receptors CX3CR1 and CCR2 play pivotal roles in monocyte recruitment to inflammatory sites, for example at the onset and progression of atherosclerosis^16–18^. Several studies assessed monocytic expression of CCR2 in ESRD patients. For example, a study by Sherry *et al*. involving children and adolescents on dialysis reported on decreased monocytic CCR2 expression which was restricted to patients suffering from recurrent serious bacterial infections and absent in patients without infection^46^. Another study, using peripheral blood mononuclear cells (PBMCs), found an increased percentage of CCR2 positive CD14^+^ monocytes in HD patients compared to healthy controls, without examining the actual expression level by means of fluorescence intensity^47^. Of note, monocytes are activated during the process of PBMC isolation^8^, which might explain the increased expression of activation markers on isolated PBMCs. Therefore, we decided to use whole blood to assess the phenotypic profile of monocytes, and did not find any changes of CCR2 expression before and after hemodialysis. This is in agreement with the aforementioned study by Sherry and coworkers, since patients suffering from acute infections were excluded in our study.

To our knowledge, we are the first to study chemokine receptor CX3CR1 protein expression on distinct monocyte subpopulations in hemodialysis patients, where we found it to be significantly impacted by uremic conditions and dialysis procedure. We observed increased levels of CX3CR1 on Mo1 monocytes and decreased levels on Mo2 and Mo3 monocytes before dialysis. The dialysis treatment led to a significant reduction of CX3CR1 expression in all monocyte subpopulations. Hence, the increase of CX3CR1 on Mo1 monocytes observed before dialysis was ablated, whereas its reduction on Mo2 and Mo3 monocytes was further enhanced after the dialysis procedure. In patients with hemolytic uremic syndrome a depletion of circulating CX3CR1 positive monocytes was reported, possibly due to glomerular infiltration by CX3CR1^+^ macrophages^48^. This might also explain our results: monocytes high in CX3CR1 expression might adhere to the activated endothelium in the course of the dialysis session, thus withdrawing them from the circulation. Our finding that less circulating leukocytes adhere to cell culture plates after a dialysis session supports this hypothesis. CKD has been linked to increased leukocyte adhesion to activated endothelium^49^. Renal replacement therapy leads to acute endothelial activation and damage^50^ and activated endothelial cells are reported to express higher levels of CX3CL1/fractalkine (FKN), which is the only known ligand of CX3CR1^51,52^. Thus, non-classical monocytes with high CX3CR1 expression might be removed from the circulation by binding to activated/damaged endothelial cells.

Schepers *et al*. conducted a transcriptome analysis of isolated monocytes from HD patients and found an increase of CX3CR1 mRNA which positively correlated with serum CRP^27^. Although results found on the transcriptional level do not necessarily mirror protein expression levels, this is in agreement with our observation that CX3CR1 protein expression is increased before dialysis on Mo1 monocytes, which represent the majority of total monocytes. Decreased CX3CR1 mRNA levels in whole blood from critically ill patients was associated with increased mortality^53^ and CX3CR1 expression on mRNA and protein level was shown to be down-regulated on circulating monocytes from sepsis patients and correlated with patient survival^54^. Therefore, monitoring of CX3CR1 was proposed as molecular biomarker to evaluate the survival probability of patients from the intensive care unit^53^.

Supporting our *in vivo* findings, we observed that incubation with foreign serum leads to a decrease of CX3CR1 surface expression on monocytes, which is significantly stronger after stimulation with uremic compared to non-uremic control serum. Similarly, LPS stimulation has been shown to result in internalization of CX3CR1 in peritoneal macrophages^55^ and downregulation of both mRNA and protein levels in human monocytes^54^, explaining, at least in part, the immunoparalysis observed under septic conditions and even the associated increased mortality^53^. CX3CR1 plays a pivotal role for a number of monocytic functions, like patrolling vessel walls, adhering to endothelium and phagocytosis^56^. A decreased phagocytic capacity has been shown for monocytes of hemodialysis patients, especially in the case of polysulphone membrane based dialysis^57^. Although proinflammatory Mo2 and Mo3 monocytes are upregulated in hemodialysis patients, decreased expression of CX3CR1 on these cells may contribute to the uremia-related impaired immune response^44^. Non-classical monocytes can respond to viral stimulation by producing pro-inflammatory cytokines^9^. They express high levels of CX3CR1, which is thought to be a marker of maturation allowing them better infiltration and adherence to the endothelium^58^. Dialysis patients have an impaired immune response to all infective agents, viruses being among them^59^. Therefore, decreased expression of CX3CR1 on intermediate and non-classical monocytes of dialysis patients may be a sign of impaired maturation and dysfunctional status leading to the aforementioned problematic immune response in dialysis patients. In support of this, LPS stimulation resulted in a diminished response of HD patients’ monocytes. Moreover, Mo2 and Mo3 monocytes from blood obtained after dialysis lost the ability to respond with significant up-regulation of TLR2, TLR4, CD18 and activated CD11b upon LPS stimulation, indicating a state of immunoparalysis especially after the dialysis procedure.

One limitation of our study is the relatively small cohort size of 15 HD patients. The differences regarding dialysis vintage, co-morbidities and dialysis membrane could at least partially explain the discrepancies among our and previous reports. The strengths of our study are the utilization of whole blood, the diifferentiation of the three monocyte subpopulations with simultaneous assessment of functionally diverse markers and a uniform use of dialysis membranes.

In conclusion, we observed a skewed distribution of monocyte subpopulations from HD patients towards proinflammatory CD16^+^ Mo2 and Mo3 monocytes, which remained unaffected by the dialysis session. The steady state of HD patients was characterized by altered phenotypes of Mo1, Mo2 and Mo3 monocytes. The dialysis procedure further enhanced these phenotypic discrepancies and monocytes obtained after dialysis show functional impairment. Moreover, our results indicate that especially monocytic CX3CR1 expression is heavily impacted by uremic conditions and might be best suited as biomarker for distinguishing between changes due to uremia and dialysis procedures.

## Material and Methods

### Patients

The study was approved by the Magdeburg Medical University ethics committee (05/15) and all methods were carried out in accordance with the relevant guidelines and regulations. HD patients from the outpatient chronic HD program of the KfH Magdeburg and healthy volunteers from the blood bank Magdeburg were enrolled in the study following informed written consent. All patients and healthy controls did not exhibit clinical signs of acute bacterial and viral infections, active malignancies and did not take immunosuppressive medication. Patients were for at least 3 months on regular hemodialysis treatment (4.0 ± 2.8 years, range: 0.4–9.4). 5 patients were diagnosed with diabetes mellitus type II. All patients were dialyzed with the polysulfone-based high-flux membrane Helixone (FX series, Fresenius Medical Care, Germany) against bicarbonate-buffered dialysate. Eleven patients had an arteriovenous fistula and four a tunneled cuffed catheter. Three patients were treated with low molecular weight fractionaled heparin, the remainder with unfractionated heparin. Clinical data, including age, gender and medical history were obtained from medical records.

### Flow Cytometric Analysis

EDTA anticoagulated blood samples from healthy donors and HD patients were collected, from the latter immediately prior and at the end of a dialysis session. Monocytes and monocyte subsets were identified by flow cytometry (BD FACSCanto II) through fluorochrome-conjugated monoclonal antibodies CD14-BV510 and CD16-PerCP-Cy5.5 (M5E2 and 3G8, Biolegend, London, UK). Antibodies CD66b-FITC (REA306, Miltenyi Biotec, Germany), HLA-DR-BV421 or –APC-Cy7 and CD86-PE (L243 and IT-2.2, Biolegend, London, UK) were used for exclusion of neutrophils and pre-gating (depicted in Supplementary Fig. 1). In three different panels, antibody combinations including CD11b-APC-Cy7 and CD163-BV421 (M1/70 and GHI/61, Biolegend, London, UK), CCR2-APC-Vio770, CD18-FITC, CD36-PE-Vio770, CX3CR1-PE-Vio770 or -PE and TLR2-PE-Vio770 (REA264, TS1/18, AC106, 2A9-1 and REA109; all Miltenyi, Biotec, Germany), were employed to assess expression of surface markers (Supplementary Fig. 1). The data were analyzed with FlowJo software (version 10.3; Tree Star, Ashland, OR, USA). The expression of surface markers was evaluated by calculating the median fluorescence intensity (MFI). Spectral spillover was corrected by creating a compensation matrix generated with the help of single antibody stainings and fluorescence minus one (FMO) control samples. All cytometer settings were saved as experiment specific application settings. Routine cytometer performance tracking was conducted according to the manufacturers’ guidelines (Becton Dickinson).

### Stimulation of whole blood with serum

Freshly drawn EDTA blood from healthy volunteers with blood group 0 was diluted 1:1 with either self-serum, non-uremic control serum or HD patient serum collected before a midweek dialysis session and incubated in 96-well cell culture plates (Cellstar, Greiner Bio-One, Germany) for 4 h at 37 °C, 5% CO_2_. Subsequently, samples were harvested and stained for FACS analysis as described above.

### Adhesion assay

Freshly drawn EDTA blood from healthy volunteers and dialysis patients before or after a dialysis session was incubated in 24-well cell culture plates (Cellstar, Greiner Bio-One, Germany) for 30 min at 37 °C, 5% CO_2_. Non-adherent cells were removed by gently washing the plate 4 times with sterile PBS to avoid disturbance of adherent cells. Pictures of each well were taken with a Zeiss microscope at 5x magnification (Zeiss Axiovert 40 CFL) and the cells per visual field were counted manually by two independent persons in a blinded manner.

### LPS stimulation

Freshly drawn EDTA blood from healthy volunteers and dialysis patients before or after a dialysis session was incubated in a 24-well cell culture plate (Cellstar, Greiner Bio-One, Germany) for 30 min at 37 °C, 5% CO_2_ with or without the addition of LPS (10 ng/ml). Subsequently, samples were harvested and stained for FACS analysis as described above. To assess monocytes responsiveness towards LPS stimulation, two additional antibodies directed against TLR4-BV421 and CD11b(activated)-PE-Cy7 (HTA125 and CBRM1/5, BioLegend, London, UK) were used. The CBRM1/5 antibody recognizes an activated form of human CD11b^60^.

### Statistical analysis

All values are depicted as mean ± standard deviation. Statistical analysis was performed using softwares GraphPad Prism 7.03 (GraphPad Software, San Diego, CA, USA) and IBM SPSS Statistics 24 for Windows (Armonk/IBM Corp., New York, NY, USA). Differences between healthy controls and HD patients were analyzed using the Mann-Whitney U-test for non-parametric variables, two-tailed. Differences between data obtained before (pre) and after (post) dialysis were calculated using the Wilcoxon matched-pairs signed rank test. P-values of < 0.05 were considered statistically significant.

## Electronic supplementary material

Supplementary File

## Data Availability

The datasets generated during and/or analysed during the current study are available from the corresponding author on reasonable request.
