# Supplementary material for: Hemodialysis-related changes in phenotypical features of monocytes
Source: Sci Rep. 2018 Sep 18;8:13964. doi: 10.1038/s41598-018-31889-2 (PMC6143543; doi:10.1038/s41598-018-31889-2)
Supplement: Supplementary file 1 — Supplementary File [file 41598_2018_31889_MOESM1_ESM.docx]

**Hemodialysis-related changes in phenotypical features of monocytes**

Vassilios Liakopoulos^1,2^, Andreas Jeron^3^, Aneri Shah^1^, Dunja Bruder^3,4^, Peter R. Mertens^1^, Xenia Gorny^1#^

^1^Clinic of Nephrology and Hypertension, Diabetes and Endocrinology, Otto-von-Guericke-University Magdeburg, Leipziger Str. 44, 39120 Magdeburg, Germany ^2^Division of Nephrology and Hypertension, 1^st^ Department of Internal Medicine, AHEPA Hospital, School of Medicine, Aristotle University of Thessaloniki, Thessaloniki, Greece

^3^Infection Immunology, Institute of Medical Microbiology, Infection Control and Prevention, Otto-von-Guericke University, Magdeburg, Germany

^4^Immune Regulation, Helmholtz Centre for Infection Research, Braunschweig, Germany

**^#^To whom all correspondence should be addressed at:**

Clinic of Nephrology and Hypertension, Diabetes and Endocrinology, Otto-von-Guericke-University Magdeburg, Leipziger Str. 44, 39120 Magdeburg, Germany

Phone: +49-391-6724703

Fax: +49-391-6715440

e-mail: xenia.gorny@med.ovgu.de

**Supplementary Figures**

**Supplementary Figure 1: Gating strategies employed in the study.**

Rows of consecutive dot plots show the gating strategy employed for each of the three antibody panels to identify monocytes and monocyte subsets in order to assess surface marker expression. Side scatter area (SSC-A) against forward scatter area (FSC-A) were used to exclude cell debris. FSC-H vs FSC-A plots were used to exclude doublets. SSC and FSC axes are linear, other axes are biexponential. Antibodies and fluorochromes are indicated.

**
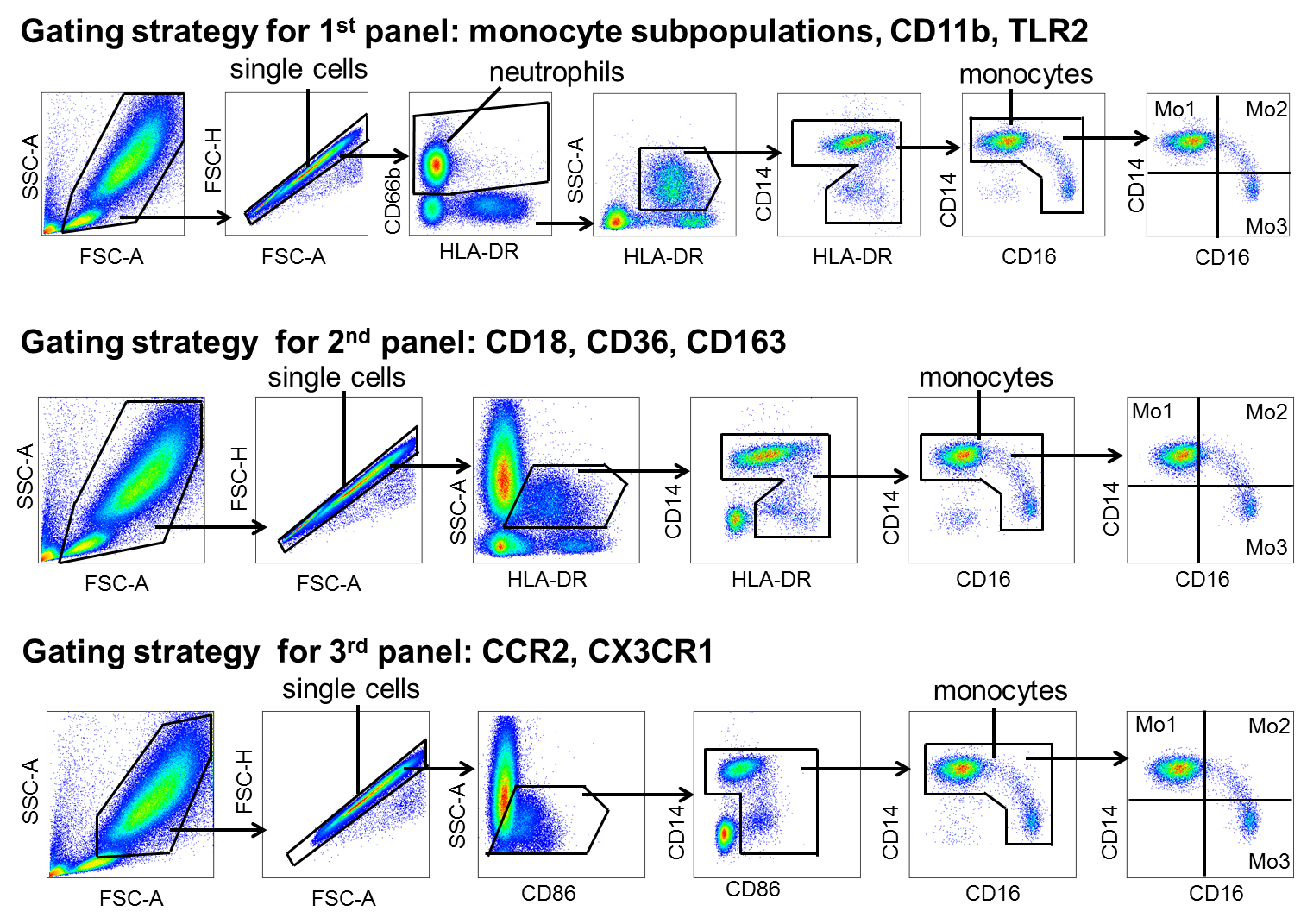
**

**Supplementary Figure 2: Total monocytes and monocyte subset distribution after 4h incubation of healthy leukocytes with control or HD patient serum.**

Dot plots show total monocytes and monocyte subset distribution of the control blood samples before incubation (CON pre incubation), after 4h incubation with self serum (CON post incubation) and after incubation with control or HD patient serum, as indicated.

**
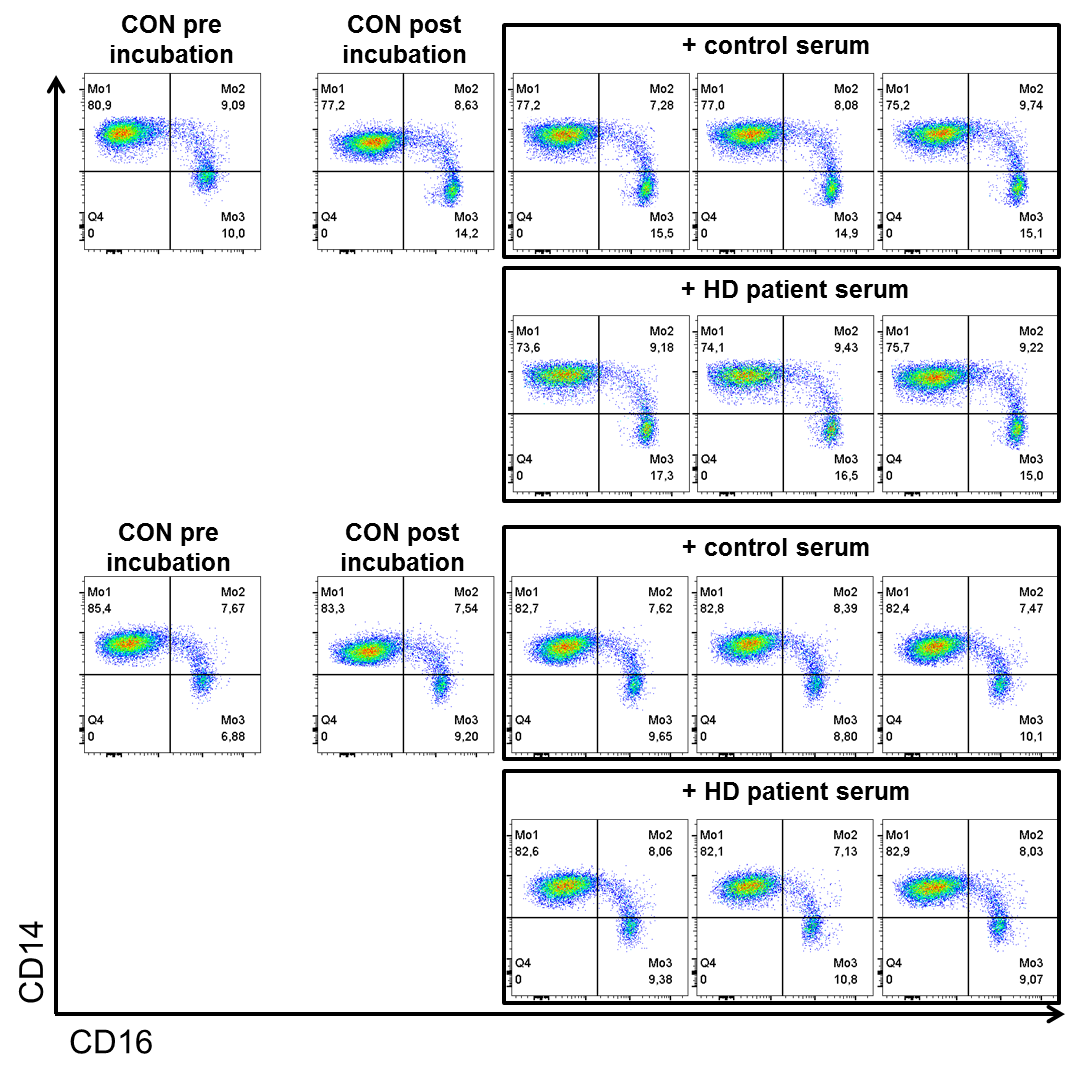
**

**Supplementary Figure 3: Changes observed after incubation of healthy leukocytes with control or HD patient serum.**

Graphs depict the percentual changes of the distribution of monocyte subpopulations, or the Median Fluorescence Intensities (MFI) of CD18, CD36 and CD163 in Mo1, Mo2 and Mo3 after incubating whole blood of healthy donors with control or HD patient serum compared to incubation with self-serum.


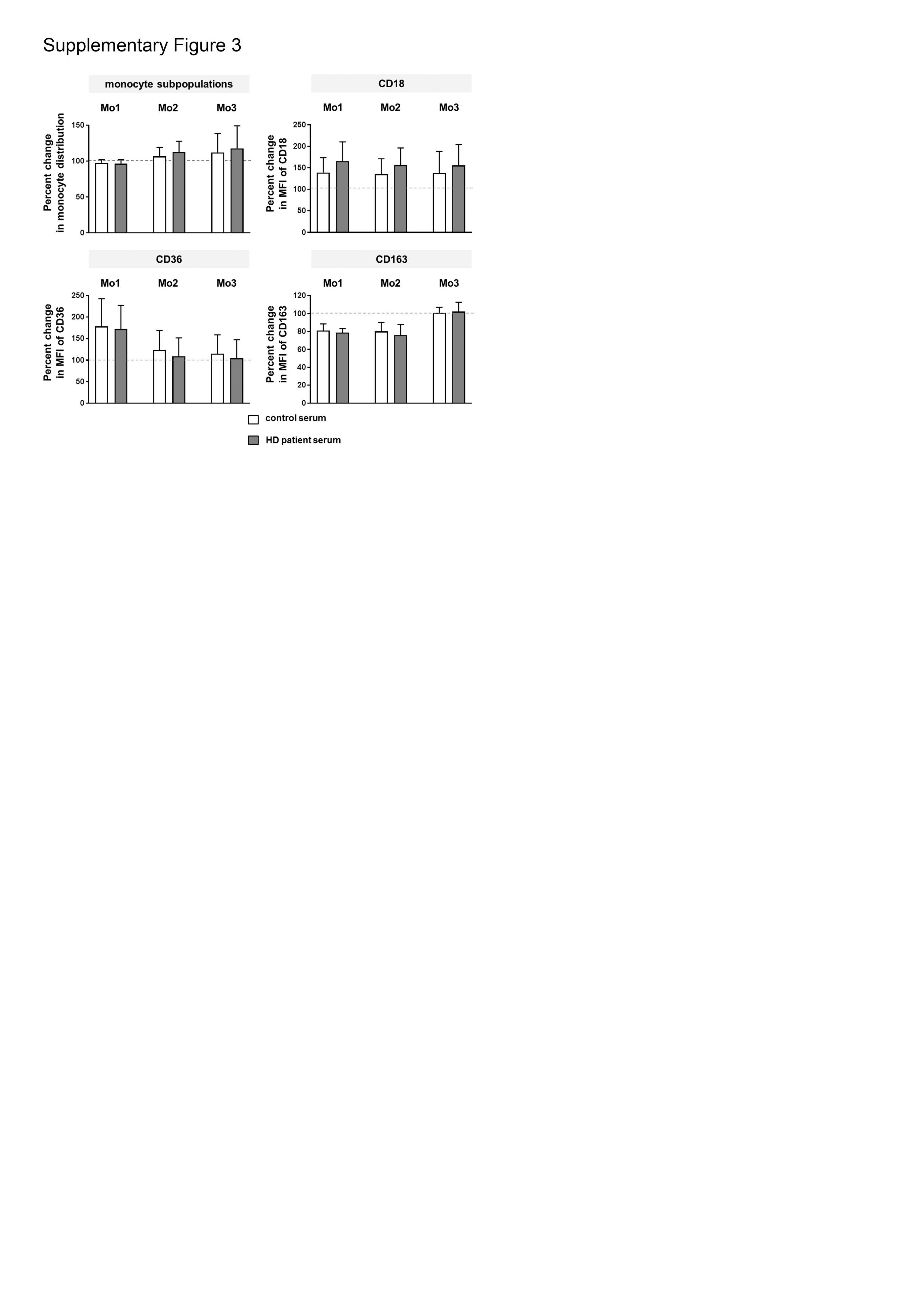


**Supplementary Figure 4: Response of healthy controls’ or HD patients' monocytes upon LPS stimulation**

Whole blood from healthy donors and HD patients was incubated for 30 min with 10 ng/ml LPS (+) or left untreated (-). The graphs show the expression of TLR2 (A), TLR4 (B), CD18 (C) or CD11b activated (D) on all three monocyte subpopulations in healthy volunteers (CON, blank circles), or HD patients before (HD pre, black circles) and after (HD post, black diamonds) a single dialysis session. P-values comparing MFI with or without LPS were calculated using the Wilcoxon matched-pairs signed rank test. MFI: median fluorescence intensity. n.s.: not significant.


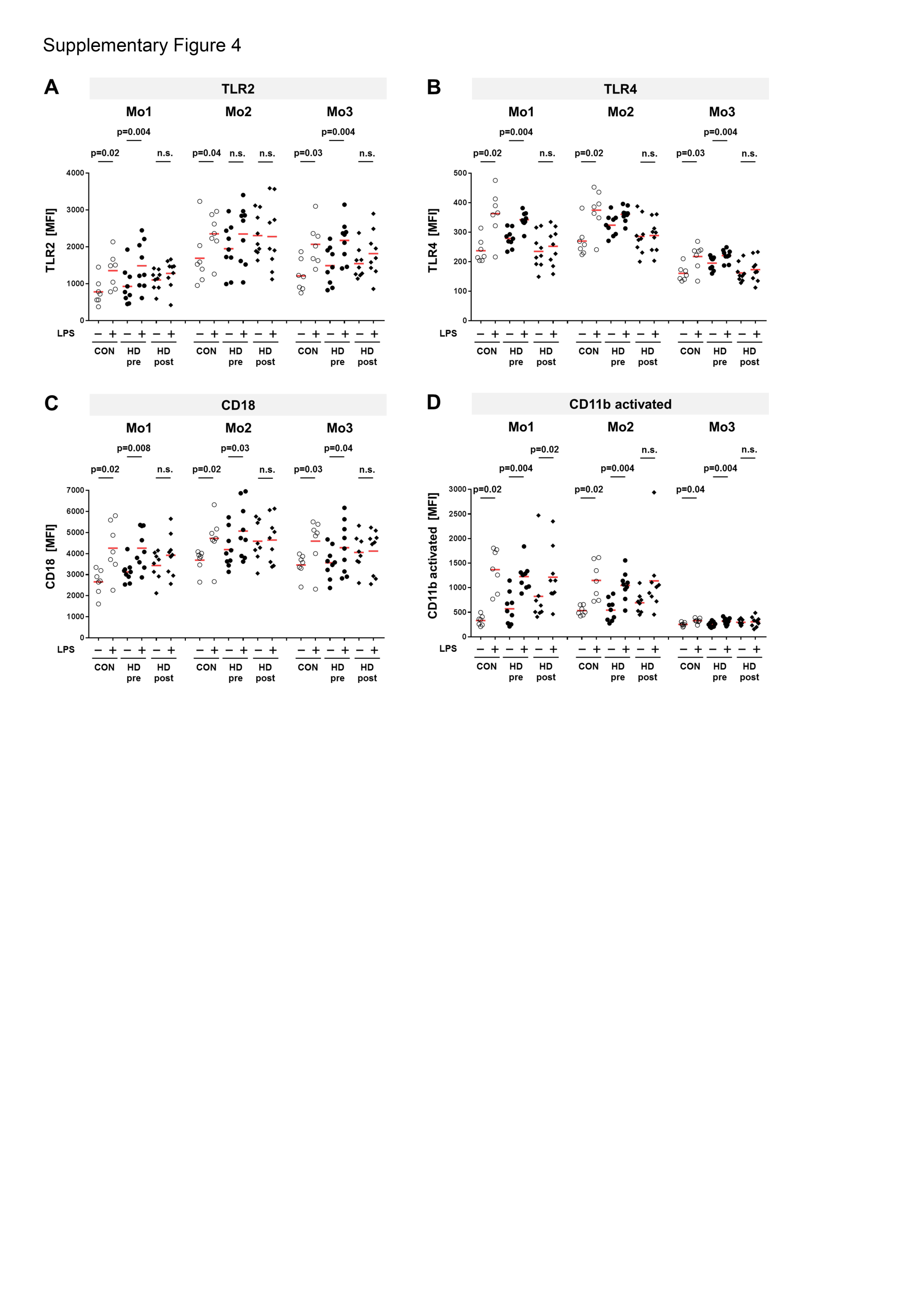


**Supplementary Table 1: Demographic and clinical data of the healthy (CON) and dialysis (HD) cohort.**

| **parameter** | **healthy cohort**  **(CON)** | **hemodialysis cohort (HD)** |
| --- | --- | --- |
| number of subjects | 16 | 15 |
| age [years] (range) | 55.8±6.3 (47-67) | 64.1±17.4 (30-84) |
| gender female/male, n | 5/11 | 5/10 |
| years on HD (range) | - | 4.0±2.8 (0.4-9.4) |
| catheter/shunt | - | 3/12 |
| leukocytes per µl (mean±SD) | 6180±1477 | 6927±1502 |
| monocytes per µl (mean±SD) | 528±179 | 661±240 |
| diabetes | - | 5/15 |
| polyneuropathy | - | 3/15 |
| coronary artery disease | - | 9/15 |
| arterial occlusive disease | - | 3/15 |
| chronic obstructive lung disease | - | 1/15 |
